# Alveolar Soft Tissue Sarcoma in the Right Thigh: A Case Study at King Abdulaziz Medical City, Jeddah, Saudi Arabia

**DOI:** 10.7759/cureus.49547

**Published:** 2023-11-28

**Authors:** Mohsin Ali, Yasir O Marghalani, Mudhawi Alhiniah

**Affiliations:** 1 Family Medicine, King Saud Bin Abdulaziz University for Health Sciences, King Abdulaziz Medical City, Jeddah, SAU; 2 College of Medicine, King Abdullah International Medical Research Center, Jeddah, SAU; 3 College of Medicine, King Saud Bin Abdulaziz University for Health Sciences, King Abdulaziz Medical City, Jeddah, SAU; 4 Medicine, King Abdulaziz University, Jeddah, SAU

**Keywords:** pd-l1 inhibitors, pembrolizumab, tyrosine kinase inhibitor (tki), surgical excision, surgically targeted radiotherapy, cancer immunotherapy, myogenic determination factor 1, transcription factor e3, alveolar soft-part sarcoma, soft-tissue sarcoma

## Abstract

The term *soft tissue sarcoma* (STS) refers to a rare group of multiple subtypes of cancer that arise in connective tissues, such as fat, muscles, and blood vessels. The disease is known to metastasize rapidly. Herein, we report a case of a 24-year-old female who complained of a painless mass in her right thigh that was gradually growing in size. The patient had lost 11 kg of weight unintentionally. On examination, there was a large mass at the right upper lateral thigh, which was warm and nontender on palpation with relatively well-defined margins clinically. The magnetic resonance imaging (MRI) scan suggested the presence of sarcoma. When biopsied, the histopathological assessment showed neoplastic infiltrates consistent with alveolar soft-part sarcoma (ASPS). There was no evidence of metastasis on computerized tomography (CT). Treatment with preoperative radiation followed by surgery was offered after discussion at the Tumor Board meeting, but the patient opted for surgery alone. This was mainly due to her concerns about the adverse effects of radiotherapy on her fertility. The patient did not develop any postoperative complications. This case highlights the importance of identifying and managing such cases promptly to improve clinical outcomes and aims to contribute to improving understanding of this rare disease.

## Introduction

The term soft tissue sarcoma (STS) refers to a group of approximately 50 different subtypes of cancer that arise in connective tissues, such as fat, muscle, and blood vessels. STS is a relatively rare type of cancer. Although STS can appear anywhere, the extremities, chest, and abdomen predominate as sites of occurrence. Surgery, radiotherapy, and chemotherapy are frequently utilized when treating such cases [1]. 

In a case report from China that was published in 2020, a six-year-old girl was diagnosed with alveolar soft-part sarcoma (ASPS) in her right lower extremity [2]. A similar case was reported in Maharashtra in 2020, where a 38-year-old woman presented with low back pain that spread to both buttocks and lower limbs, and she was diagnosed with spine and hip ASPS [3]. In another case report published in 2022, a 25-year-old woman presented with proptosis and redness of the right eye and was diagnosed with ASPS of the superior rectus muscle [4].

In this case report, we present an interesting case of a 24-year-old female patient who complained of a painless mass in the right upper thigh that was gradually increasing in size, which was associated with weight loss. She was diagnosed with ASPS; however, the disease had not metastasized.

## Case presentation

A 24-year-old female came to the Employee Health Clinic for the first time with a complaint of a painless mass at her right thigh measuring 4 cm x 3 cm that had been gradually growing for the past six months. The mass was bothering her while changing position from sitting to standing. The review of systems revealed nothing unusual aside from the unintentional weight loss of 11 kg over the past six months from her baseline weight of 73 kg (body mass index [BMI] 28.3). There were no neurological, respiratory, gastrointestinal, urinary tract, or musculoskeletal symptoms. There were no known close relatives with any history of cancer.

On examination, the right thigh was warm to palpate at the site of the lesion. A 4 cm x 3 cm nontender soft tissue mass was palpable at the right upper lateral thigh, which seemed to be attached to the muscle underneath. She was immediately referred to the Orthopedics Department, where an MRI scan and biopsy were organized. Meanwhile, the patient opted to have an ultrasound scan at another institution to expedite the process, which suggested the possibility of STS.

**Figure 1:**
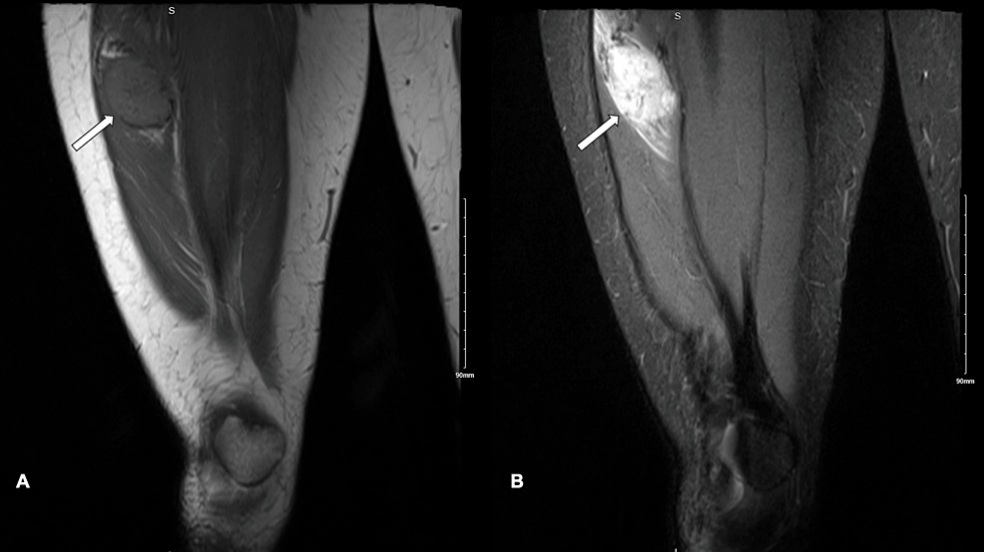
Coronal MRI of the right thigh: (A) T1; (B) T2; arrow: pointing to the lesion of interest. MRI, magnetic resonance imaging

**Figure 2:**
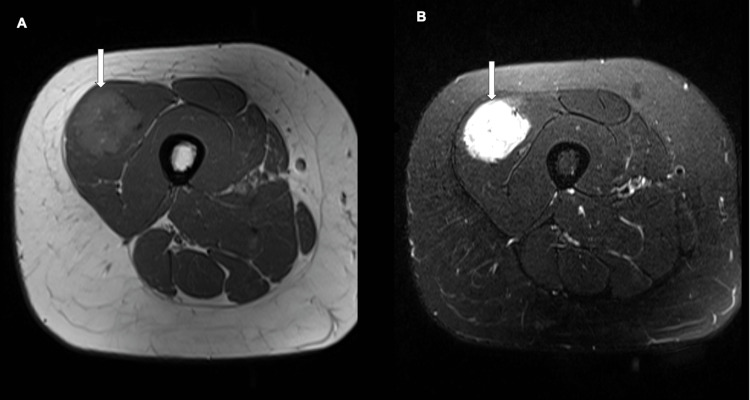
Axial MRI of the right thigh: (A) T1; (B) T2; arrow: pointing to the lesion of interest. MRI, magnetic resonance imaging

MRI showed an intramuscular mass measuring 4.1 cm x 3.5 cm x 3.3 cm in the lateral aspect of the quadriceps, mainly in the vastus lateralis. The mass appeared round with well-defined borders and solid texture. Regarding signal intensities, the mass appeared slightly hyperintense to muscles on T1 and hyperintense on T2. Moreover, it showed homogeneous enhancement after contrast infusion along with visible supplying and draining vessels. The other muscles, subcutaneous fat, and femur displayed normal morphology and signal intensity. In addition, there was no evidence of regional lymphadenopathy (Figures 1-2).

The biopsy demonstrated a neoplastic infiltrate consistent with ASPS. The patient underwent a CT scan of the abdomen, chest, and pelvis for staging, which showed no evidence of metastases.

The patient's case was discussed at the Tumor Board meeting. There was consensus among the members that pre-op radiation should be done because of the disease's inherent risk of spreading quickly; however, the patient opted to undergo surgical intervention only, because she was concerned about the adverse effects of radiotherapy that may have on her fertility in the future. The patient was referred to a more specialized surgical branch of the institution in Riyadh, where the surgery was performed. She got pregnant one year after the surgery and delivered a healthy child after nine months without any complications. She remains disease-free, with no signs of recurrence three years post-resection, and has also fully regained her weight loss.

## Discussion

ASPS is a rare and unique tumor that mainly affects young people. The prognosis is poor and is frequently characterized by early metastases despite a relatively indolent clinical course [5]. A database search of the United States population between 1973 and 2014 reported only 267 cases [5].

Less than 1% of all STSs are ASPSs, which is a rare and distinctive STS subtype [5]. Only 267 patients with this diagnosis were found in a surveillance, epidemiology, and results database search in the United States population between 1973 and 2014 [6]. Before the age of 30, there is a female predominance with a reverse ratio for older ages [7]. In young female patients, ASPS most commonly manifests as a painless mass [5,6]. Christopherson et al. first described ASPS in 1952 as an entity with distinct clinical and pathologic features [7]. The imbalanced recurring t(X;17)(p11;q25) translocation, which results in a chimeric ASPSCR1-transcription factor E3 (TFE3), is a hallmark of ASPS [8,9]. Patients between the ages of 15 and 35 are more likely to develop ASPS [8,9]. Histopathologic analysis using morphology and immunohistochemistry is used to make the diagnosis, which can be difficult [10]. ASPS has a high rate of distant metastasis, with a greater propensity for lungs, bones, and brain; however, it is considered a relatively slow-growing disease [11].

There are ongoing clinical trials involving the use of immunologic therapy such as Checkpoint Inhibitors, for example, atezolizumab, nivolumab, and sunitinib, due to its theorized pathogenesis involving gain-of-function fusion proteins [12]. Surgery remains the mainstay of treatment currently [13].

Clinicians need to remain open-minded about the possibility of sarcoma even in young patients. The mass felt warm on palpation at presentation, which is an important sign in identifying such cases, possibly due to the high metabolic rate of these lesions. However, this is only a speculation and more information from other cases is required to reach such a conclusion. Positive outcomes are achievable even without the use of radiotherapy. The patient got pregnant after the surgery and delivered a child without any complications. The use of radiotherapy in cases of childbearing age needs to be carefully considered due to potential negative implications on the prospects of pregnancy.

## Conclusions

One highlight of this case report is that anyone presenting with soft tissue mass, even at a younger age, with red flags, that is, progressively getting bigger with unintentional weight loss should be urgently investigated for suspected sarcoma. The second highlight is the positive outcome, that is, no recurrence after three years of surgical correction achieved without the use of radiotherapy, with surgery as the mainstay of treatment. The patient got pregnant one year after the operation and delivered a child without any complications. One limitation of this case report is its susceptibility to publication and selection bias in terms of presentation and management.

Due to the scarcity of the literature covering such a rare disease, reporting of this condition's clinical presentation, its management plan, and clinical outcomes will aid other clinicians in decision-making.
